# Vitamin E Improves Cellular and Structural Bone Histomorphometry in an Alcohol-Induced Osteoporosis Rat Model

**DOI:** 10.3390/ph17121730

**Published:** 2024-12-20

**Authors:** Norazlina Mohamed, Seham Salem Ahmed Abukhadir, Syed Alhafiz Syed Hashim, Nur Sabariah Adnan, Muhamad Arizi Aziz, Norliza Muhammad

**Affiliations:** 1 Department of Pharmacology, Faculty of Medicine, Universiti Kebangsaan Malaysia, Cheras, Kuala Lumpur 56000, Malaysia; sehams2020@yahoo.com (S.S.A.A.); syedalhafiz@ukm.edu.my (S.A.S.H.); sabariahadnan@gmail.com (N.S.A.); arzarz81@gmail.com (M.A.A.); norliza_ssp@hctm.ukm.edu.my (N.M.); 2 Institute of Pharmaceutical Science, King’s College London, Franklin-Wilkins Building, 150 Stamford Street, London SE1 9NH, UK

**Keywords:** alcohol-induced osteoporosis, alpha-tocopherol, palm vitamin E, bone histomorphometry

## Abstract

**Background**: Alcohol-induced osteoporosis is a significant health concern, impairing bone formation and enhancing resorption, thereby weakening skeletal integrity. This study examines the effects of palm vitamin E on bone histomorphometry in a male rat model of alcohol-induced osteoporosis. **Methods**: Three-month-old Sprague–Dawley rats were randomized into seven groups, with one baseline control group (BC) and six experimental groups undergoing a two-phase treatment. In the first month, the control group received normal saline, while experimental groups received intraperitoneal alcohol (3 g/kg) three times weekly. For the subsequent two months, alcohol treatment continued in one group (A), while others received olive oil (C), saline (AN), alpha-tocopherol (AA), or palm vitamin E (AE) orally. **Results**: Femur histomorphometric analysis post-sacrifice showed that alcohol exposure significantly decreased osteoblastic activity and impaired bone microarchitecture, evidenced by reduced Ob.S/BS, OS/BS, OV/BV, Tb.Th, BV/TV, and Tb.N, alongside increased Oc.S/BS, ES/BS, and Tb.Sp. Both alpha-tocopherol and palm vitamin E improved bone parameters, with palm vitamin E showing superior efficacy except in OV/BV. **Conclusions**: These findings suggest that palm vitamin E may offer a therapeutic benefit for mitigating alcohol-induced bone damage.

## 1. Introduction

Alcohol consumption poses a significant threat to bone health. Its abuse is linked to a high incidence of fractures and a spectrum of related complications, including infections, nerve damage, and vascular injuries. Numerous studies have demonstrated alcohol consumption as a pivotal risk factor for osteoporosis [1,2]. It heightens the risk of fractures, impedes fracture healing [3,4] and is frequently linked with elevated mortality rates [5]. Excessive alcohol consumption is a significant public health concern, particularly among adolescents and young adults, where prevalent patterns like binge drinking are common [6]. According to the World Health Organization’s 2024 report, in 2019, 17% of individuals aged 15 and older engaged in heavy episodic drinking (binge drinking). Among 15–19-year-olds, the prevalence of alcohol consumption was notably high at 22%, showing very little gender disparity and a concerning trend of increasing rates from initially low levels in certain regions [7]. Binge drinking poses various risks, particularly to bone health, especially when occurring during the critical phase of peak bone mass (PBM) attainment during adolescents and young adults. This period is crucial for bone development and strength [8], and disruptions due to alcohol consumption can impair bone growth and increase the risk of osteoporosis later in life.

Osteoporosis is typically defined as low bone mineral density, which is assessed using the gold standard dual-energy X-ray absorptiometry scan. A BMD score below −2.5 indicates osteoporosis. Alcohol-induced osteoporosis is attributed to various mechanisms, including oxidative stress and impairment to osteogenic processes. Alcohol not only stimulates the generation of reactive oxygen species (ROS), notorious for inducing cellular damage, but also enhances cytochrome P450 activity, which further promotes ROS production [9]. Moreover, alcohol consumption is associated with adverse effects on liver function [10], which can impair the generation of antioxidative defenses, such as superoxide dismutase, and glutathione peroxidase [11]. While ROS from osteoclasts typically aid in calcified tissue breakdown and remodeling, excessive ROS under pathological conditions overwhelm antioxidant defenses, leading to hyperactive osteoclasts [12], thereby contributing to the development and progression of osteoporosis. Alcohol impairs the osteogenic process by hindering the differentiation of mesenchymal stem cells into bone cells. This inhibition occurs due to the activation of Forkhead Box Protein O-specific signaling pathways [13], leading to a disruption in bone growth and fracture healing. Moreover, alcohol disrupts Akt phosphorylation and its recruitment to the plasma membrane by upregulating PTEN. This, in turn, interferes with the Akt/GSK3β/β-catenin signaling pathway and reduces the expression of markers essential for osteogenic differentiation [13]. Chen et al. (2017) demonstrated that alcohol significantly decreased osteogenic activity in human bone mesenchymal stem cells (hBMSCs) in vitro and impaired bone formation in the rat femoral head in vivo [14].

There are eight forms of vitamin E: α, β, γ, and δ variations of tocopherols and tocotrienols [15,16]. Each form of vitamin E includes a ring with a chromanol structure and a side chain. Tocotrienols have a farnesyl side chain, while tocopherols feature a saturated phytyl side chain [17]. Tocotrienols’ unsaturated side chains enable them to better permeate the membrane lipid bilayer. Vitamin E is a crucial antioxidant for membrane lipids against peroxidation [18,19]. Tocopherols are found widely in polyunsaturated vegetable oils and the germ of cereal seeds [20], while tocotrienols are found usually in palm oil, cereal grains, and rice bran [21]. Vitamin E is mainly available commercially as alpha-tocopherol and commonly used as an antioxidant supplement [22]. Alpha-tocopherol is the most widely studied, as it exhibits the most biological activity and is more prevalent in human tissues [23] and blood [24] compared to other forms.

Numerous studies have shown that vitamin E supplementation can guard against bone loss and damage caused by stress triggered by hormone deficiency or oxygen-derived free radicals [25]. Vitamin E protects bone by enhancing antioxidant defenses against free radicals and neutralizing lipid peroxidation radicals, thereby averting oxidative damage [25]. Free radicals have been implicated in promoting osteoclastic differentiation under oxidative stress and explored as potential therapeutic targets for ROS-mediated osteoclast-related diseases [26]. Vitamin E plays a crucial role in mitigating oxidative stress by scavenging ROS through its chromanol ring, which donates hydrogen atoms to neutralize free radicals [27]. This action interrupts lipid peroxidation chain reactions, thereby protecting cellular membranes from oxidative damage. Tocotrienols, with their unsaturated side chains, integrate deeply into lipid bilayers, effectively targeting peroxyl radicals within membrane [28]. By stabilizing cellular membranes, Vitamin E prevents the accumulation of harmful lipid peroxidation-derived aldehydes, such as malondialdehyde, which can lead to cellular damage [29]. At the cellular level, Vitamin E mitigates osteoclast hyperactivity by inhibiting NF-κB signaling and reducing pro-osteoclastogenic cytokines like RANKL, thus promoting a balanced bone remodeling process [25]. Furthermore, it preserves mitochondrial membrane potential [30] and reduces caspase expression [31], thereby decreasing oxidative stress-induced apoptosis of osteocytes.

However, the effects of vitamin E on bone health are dose and isoform dependent [32]. High doses of α-tocopherol have been linked to adverse effects, including decreased bone mass. For instance, in vitro studies suggest that high doses stimulate osteoclast fusion [33], while in vivo, normal ambulatory mice supplemented with 500 IU/kg of α-tocopherol exhibited reduced trabecular number and bone volume compared to those receiving lower doses (15 or 75 IU/kg) [34]. Clinically, high doses of vitamin E (≥400 IU/day) have also been associated with increased all-cause mortality [35]. Therefore, it is crucial to carefully consider both the isoform and dosage of vitamin E.

Our previous study found that palm vitamin E could boost the activity of genes associated with bone formation in rats exposed to nicotine [36]. This understanding could potentially aid in creating a novel treatment modality for addressing metabolic bone conditions such as osteoporosis. We have also shown that both palm vitamin E and alpha-tocopherol increased bone mineral content, but only palm vitamin E was able to improve bone strength in alcohol-treated rats [37]. This current study aimed to investigate how supplementing with palm vitamin E affects cellular and structural bone histomorphometry parameters in rats with alcohol induced bone loss. The results are intended to enhance knowledge of alcohol effects on bones and demonstrate how vitamin E could potentially help to improve bone health after alcohol consumption is stopped.

## 2. Results and Discussion

### 2.1. Results

#### 2.1.1. Cellular Bone Histomorphometry

##### Osteoblast Surface/Bone Surface % (Ob.S/BS %)

**Figure 1:**
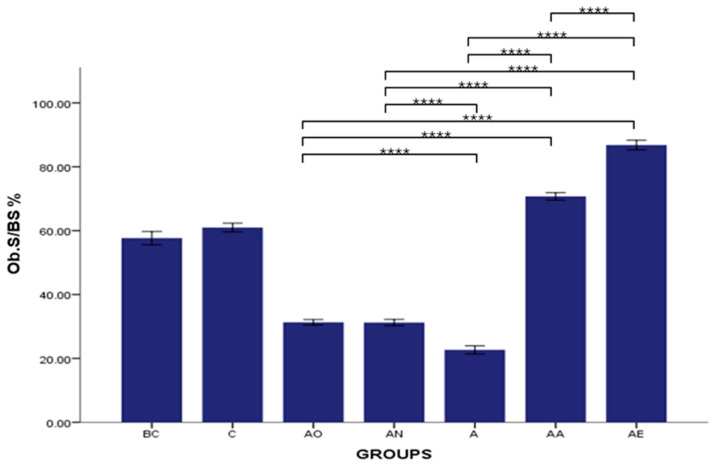
The effects of vitamin E supplementation on osteoblast surface in alcohol-induced rats. Abbreviations: BC: Baseline Control, C: Control, AO: Alcohol Olive Oil, AN: Alcohol Normal Saline, A: Alcohol, AA: Alcohol Alpha-Tocopherol, AE: Alcohol Palm Vitamin E. Ob.S/BS: Osteoblast surface/bone surface. Data are presented as mean ± SEM. **** Indicates significant difference between groups (*p* < 0.0001).

There was no significant difference in the mean Ob.S/BS % between the Baseline Control (BC) and Control (C) groups. However, the mean Ob.S/BS % was significantly decreased in the Alcohol Olive Oil (AO), Alcohol Normal Saline (AN) and Alcohol (A) groups compared to the Control groups (BC and C) (*p* < 0.0001). Both the Alcohol Alpha-Tocopherol (AA) and Alcohol Palm Vitamin E (AE) groups showed a significant reversal of the alcohol-induced reduction in Ob.S/BS % (*p* < 0.0001). These groups exhibited a significantly higher mean osteoblast surface compared to both the Control (BC and C) and alcohol-treated groups (AO, AN and A) (*p* < 0.0001). Furthermore, the mean osteoblast surface in the Alcohol Palm Vitamin E (AE) group was significantly higher than that in the Alcohol Alpha-Tocopherol (AA) group (*p* < 0.0001) (Figure 1).

##### Osteoclast Surface/Bone Surface % (Oc.S/BS %)

**Figure 2:**
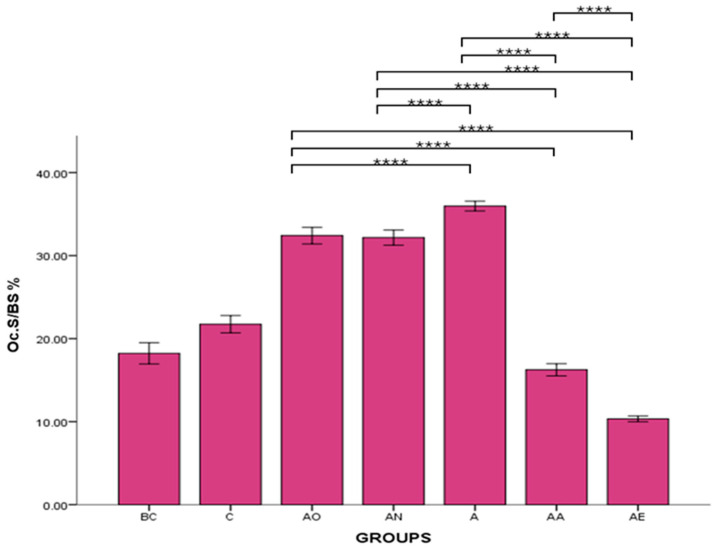
The effects of vitamin E supplementation on osteoclast surface in alcohol-induced rats. Abbreviations: BC: Baseline Control, C: Control, AO: Alcohol Olive Oil, AN: Alcohol Normal Saline, A: Alcohol, AA: Alcohol Alpha-Tocopherol, AE: Alcohol Palm Vitamin E. Oc.S/BS: osteoclast surface/bone surface. Data are presented as mean ± SEM. **** Indicates significant difference between groups (*p* < 0.0001).

There was no significant difference in the osteoclast surface between the BC and C groups. However, the mean Oc.S/BS % of AO, AN and A groups were significantly increased compared to the C group (*p* < 0.0001). Conversely, the mean Oc.S/BS % for the AA and AE groups was significantly decreased compared to the C groups (*p* < 0.0001). Palm vitamin E supplementation led to a reduction in the osteoclast surface, which was significantly different compared to AO, AN and A groups. This effect was also observed in rats treated with alpha-tocopherol. However, there was a significant difference between the AE and AA groups, with the mean Oc.S/BS % for the AE group significantly lower than that for the AA group (*p* < 0.0001) (Figure 2).

##### Eroded Surface/Bone Surface % (ES/BS %)

**Figure 3:**
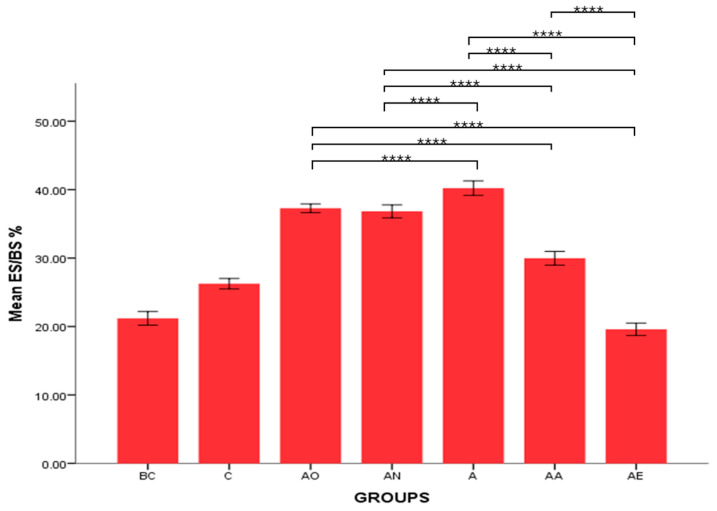
The effects of vitamin E supplementation on eroded surface in alcohol-induced rats. Abbreviations: BC: Baseline Control, C: Control, AO: Alcohol Olive Oil, AN: Alcohol Normal Saline, A: Alcohol, AA: Alcohol Alpha-Tocopherol, AE: Alcohol Palm Vitamin E. Mean ES/BS: eroded surface/bone surface. Data are presented as mean ± SEM. **** Indicates significant difference between groups (*p* < 0.0001).

**Figure 4:**
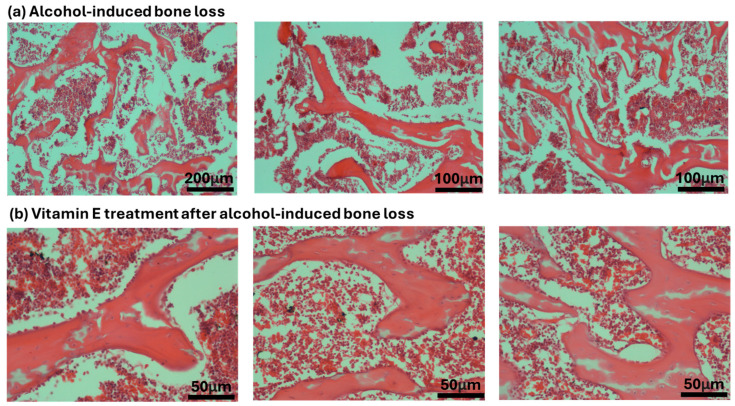
Photomicrographs of H and E stain on cellular bone histomorphometry after treatment with alcohol and vitamin E.

There was no significant difference in the mean ES/BS % between the Baseline Control (BC) and Control (C) groups. However, the mean ES/BS % of the Alcohol Olive Oil (AO), Alcohol Normal Saline (AN) and Alcohol (A) groups was significantly increased compared to the Control groups (BC and C) (*p* < 0.0001). Conversely, the mean ES/BS % for both the Alcohol Alpha-Tocopherol (AA) and Alcohol Palm Vitamin E (AE) groups was significantly decreased compared to the Control groups (BC and C) (*p* < 0.0001). Alpha-tocopherol (AA) and palm vitamin E (AE) supplementation were able to reverse alcohol’s effect and caused a significant decrease in eroded surface compared to the AO, AN and A groups. Additionally, the mean ES/BS % for the AE group was significantly decreased compared to the AA group (*p* < 0.0001) (Figure 3 and Figure 4).

##### Osteoid Surface/Bone Surface % (OS/BS %)

**Figure 5:**
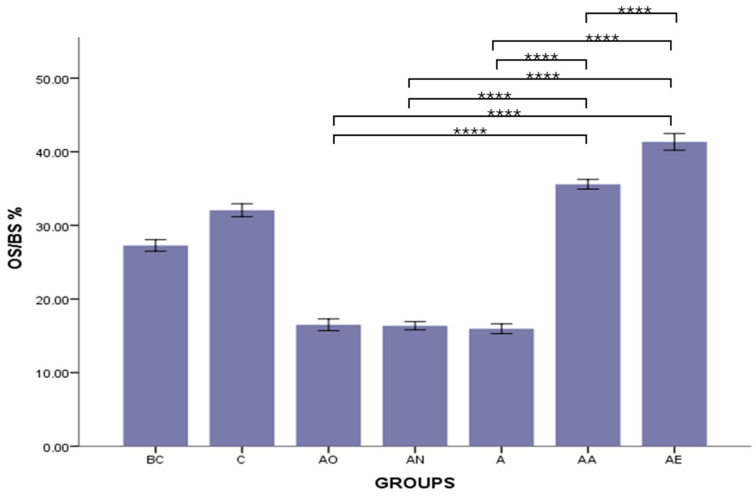
The effects of vitamin E supplementation on osteoid surface in alcohol-induced rats. Abbreviations: BC: Baseline Control, C: Control, AO: Alcohol Olive Oil, AN: Alcohol Normal Saline, A: Alcohol, AA: Alcohol Alpha-Tocopherol, AE: Alcohol Palm Vitamin E. OS/BS: osteoid surface/bone surface. Data are presented as mean ± SEM. **** Indicates significant difference between groups (*p* < 0.0001).

There was no significant difference in the mean OS/BS % between the Baseline Control (BC) and Control (C) groups. However, the mean OS/BS % of both the Alcohol Olive Oil (AO), Alcohol Normal Saline (AN) and Alcohol (A) groups was significantly decreased compared to the Control groups (BC and C) (*p* < 0.0001). Conversely, both the Alcohol Alpha-Tocopherol (AA) and Alcohol Palm Vitamin E (AE) groups had significantly higher OS/BS % compared to the Control groups (BC and C) (*p* < 0.0001). Both alpha-tocopherol (AA) and palm vitamin E (AE) supplementation were able to increase the osteoid volume in a significantly higher manner than in the alcohol groups (AO, AN and A). However, the OS/BS % for the Alcohol Alpha-Tocopherol (AA) group was still significantly lower than that for the Alcohol Palm Vitamin E (AE) group (*p* < 0.0001) (Figure 5).

##### Osteoid Volume/Bone Volume % (OV/BV %)

**Figure 6:**
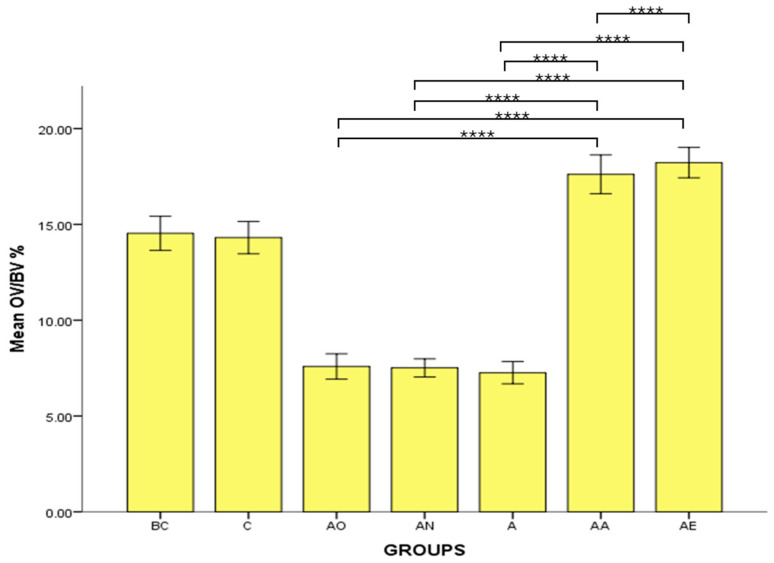
The effects of vitamin E supplementation on osteoid volume in alcohol-induced rats. Abbreviations: BC: Baseline Control, C: Control, AO: Alcohol Olive Oil, AN: Alcohol Normal Saline, A: Alcohol, AA: Alcohol Alpha-Tocopherol, AE: Alcohol Palm Vitamin E. Mean OV/BV: osteoid volume/bone volume. Data are presented as mean ± SEM. **** Indicates significant difference between groups (*p* < 0.0001).

There was no significant difference in the mean OV/BV % between the Baseline Control (BC) and Control (C) groups. However, the mean OV/BV % of the Alcohol Olive Oil (AO), Alcohol Normal Saline (AN) and Alcohol (A) groups was significantly decreased compared to the Control groups (BC and C) (*p* < 0.0001). Conversely, both the Alcohol Alpha-Tocopherol (AA) and Alcohol Palm Vitamin E (AE) groups had significantly increased OV/BV % compared to the Control groups (BC and C) (*p* < 0.0001). Alpha-tocopherol (AA) and palm vitamin E (AE) supplementation were able to increase the osteoid volume in a significantly higher manner than in the alcohol groups (AO, AN and A). However, there was no significant difference in mean osteoid volume between the Alcohol Palm Vitamin E (AE) and Alcohol Alpha-Tocopherol (AA) groups (Figure 6).

#### 2.1.2. Structural Bone Histomorphometry

##### Trabecular Thickness (Tb.Th), Bone Volume/Total Volume (BV/TV) and Trabecular Number (Tb.N)

**Figure 7:**
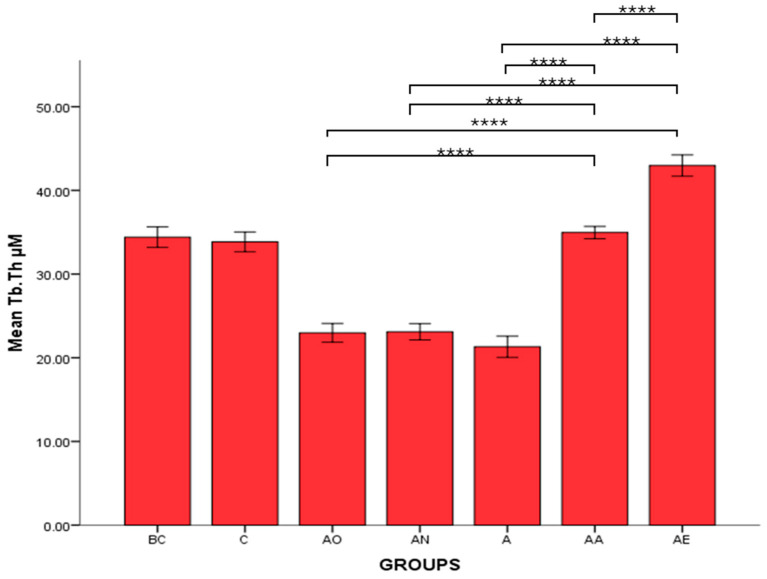
The effects of vitamin E supplementation on trabecular thickness of alcohol-induced rats. Abbreviations: BC: Baseline Control, C: Control, AO: Alcohol Olive Oil, AN: Alcohol Normal Saline, A: Alcohol, AA: Alcohol Alpha-Tocopherol, AE: Alcohol Palm Vitamin E. Mean Tb.Th: trabecular thickness. Data are presented as mean ± SEM. **** Indicates significant difference between groups (*p* < 0.0001).

**Figure 8:**
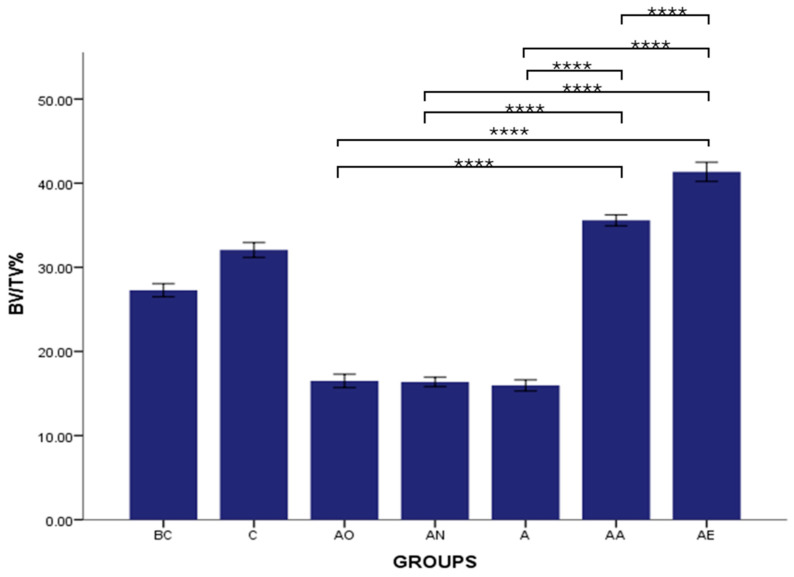
The effects of vitamin E supplementation on Bone Volume/Total Volume of alcohol-induced rats. Abbreviations: BC: Baseline Control, C: Control, AO: Alcohol Olive Oil, AN: Alcohol Normal Saline, A: Alcohol, AA: Alcohol Alpha-Tocopherol, AE: Alcohol Palm Vitamin E. Mean BV/TV: bone volume/total volume. Data are presented as mean ± SEM. **** Indicates significant difference between groups (*p* < 0.0001).

**Figure 9:**
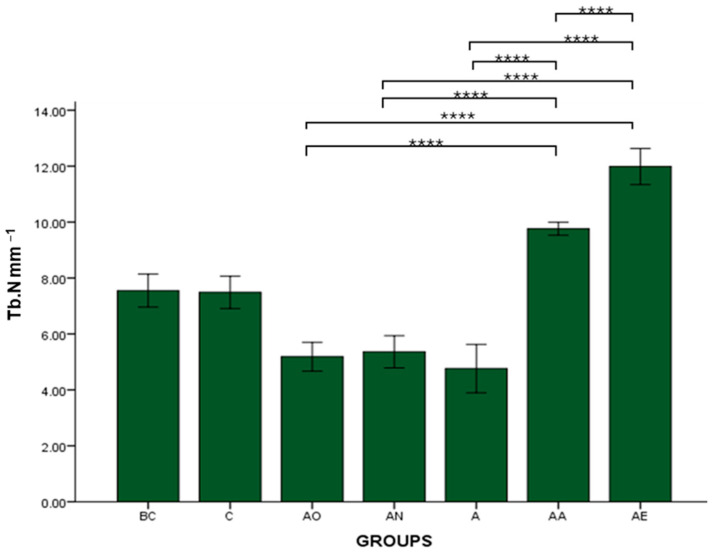
The effects of vitamin E supplementation on Trabecular Number of alcohol-induced rats. Abbreviations: BC: Baseline Control, C: Control, AO: Alcohol Olive Oil, AN: Alcohol Normal Saline, A: Alcohol, AA: Alcohol Alpha-Tocopherol, AE: Alcohol Palm Vitamin E. Mean Tb.N: trabecular number. Data are presented as mean ± SEM. **** Indicates significant difference between groups (*p* < 0.0001).

**Figure 10:**
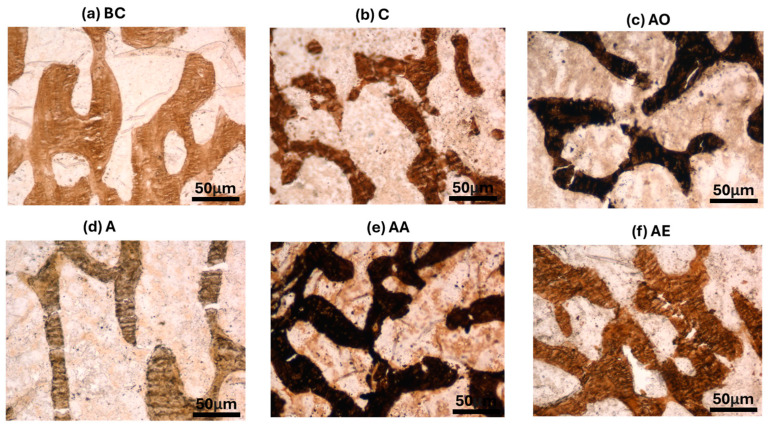
Photomicrographs of Von Kossa stain on structural bone histomorphometry after treatment with vitamin E. Abbreviation: Treatment groups: BC: Baseline Control, C: Control, A: Alcohol, AO: Alcohol Olive Oil, AA: Alcohol Alpha-Tocopherol, AE: Alcohol Palm Vitamin E.

There was no significant difference in the mean Tb.Th, BV/TV and Tb.N between the Baseline Control (BC) and Control (C) groups. The mean Tb.Th, BV/TV and Tb.N of both the Alcohol Olive Oil (AO), Alcohol Normal Saline (AN) and Alcohol (A) groups was significantly lower compared to the Control groups (BC and C) (*p* < 0.0001). Both the Alcohol Alpha-Tocopherol (AA) and Alcohol Palm Vitamin E (AE) groups had significantly higher trabecular thickness, bone volume/total volume and trabecular number compared to the Control groups (BC and C) (*p* < 0.0001). Interestingly, both alpha-tocopherol (AA) and palm vitamin E (AE) supplementation were able to increase the Tb.Th, BV/TV and Tb.N significantly compared to the alcohol groups (AO, AN and A). Between AA and AE, AE demonstrated a significant increase in Tb.Th, BV/TV and Tb.N compared to AA (Figure 7, Figure 8 and Figure 9). Trabecular thickness appears poor and highly separated in the AO groups, while the trabecular appearance is improved in the AA and AE groups (Figure 10).

##### Trabecular Separation (Tb.Sp)

**Figure 11:**
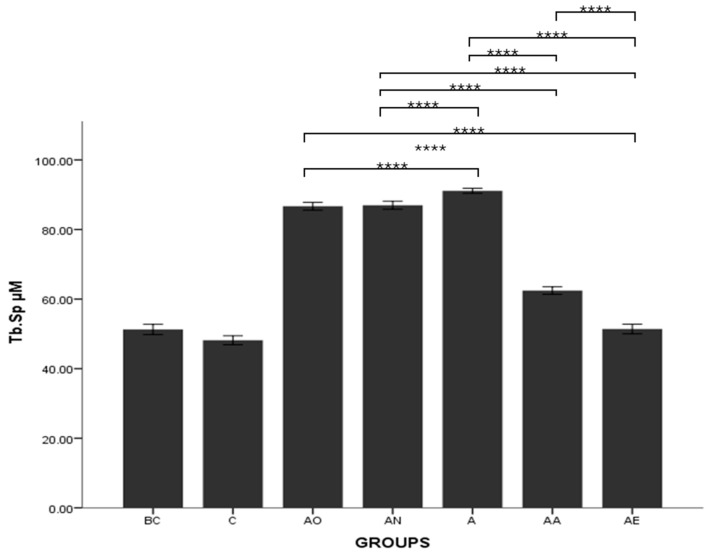
The effects of vitamin E supplementation on trabecular separation of alcohol-induced rats. Abbreviations: BC: Baseline Control, C: Control, AO: Alcohol Olive Oil, AN: Alcohol Normal Saline, A: Alcohol, AA: Alcohol Alpha-Tocopherol, AE: Alcohol Palm Vitamin E. Mean Tb.Sp: trabecular separation. Data are presented as mean ± SEM. **** Indicates significant difference between groups (*p* < 0.0001).

There was no significant difference in the osteoclast surface between the BC and C groups. The mean trabecular separation of the AO, AN and A groups was significantly higher compared to the BC and C groups (*p* < 0.0001). The mean Tb.Sp for the AA and AE groups was significantly lower compared to AO, AN and A groups (*p* < 0.0001). AE and AA groups demonstrated significant differences, with the mean Tb.Sp for AE lower than that for the AA group (*p* < 0.0001) (Figure 11).

### 2.2. Discussion

Alcohol is known to be one of the significant contributors to osteoporosis [38,39]. Despite extensive research, the precise pathological mechanisms underlying alcohol-induced osteoporosis remain inadequately understood. Prior studies have developed an animal model of alcohol-induced osteoporosis by administering ethanol intraperitoneally at a dosage of 3 g/kg [40,41] which enable researchers to closely study the effects of alcohol on bone health and potential therapeutic interventions in a controlled setting. Therefore, we based our study on the animal model mentioned. Tocotrienol has been explored as a potential therapeutic agent for restoring bone health. Tocotrienol, at a dose of 60 mg/kg body weight, administered orally, has been reported to effectively prevent bone loss in many rat models [42].

We explored bone microarchitecture via histomorphometric analysis, as this technique provides detailed quantitative data and has been extensively applied to test changes in the mass and structure of cancellous bone [43]. This approach is crucial for understanding bone health and the effects of various treatments or conditions. Alcohol consumption has been shown to significantly impair bone health by decreasing bone formation and increasing bone resorption. It leads to a reduction in bone mineral density, bone volume, trabecular thickness, and trabecular number in human bone mesenchymal stem cells through the suppression of the Wnt/β-catenin signaling pathways [14] ultimately causing osteoporosis. Although abstinence is recommended and has positive outcomes, detrimental effects of alcohol on bone can persist or be only partially resolved [44] even with alcohol abstinence [45], emphasizing the need for optimized intervention in this domain.

In this study, we demonstrated that alcohol significantly reduces osteoblastic bone formation, as evidenced by decrease in Ob.S/BS (%), OS/BS (%), and OV/BV (%), while increasing osteoclastic bone resorption, as indicated by a significant rise in Oc.S/BS (%) and ES/BS (%) within the alcohol-induced groups (AO, AN and A). We also showed that alcohol impairs bone microstructure by causing trabecular thinning, a reduction in trabecular volume and number, and an increase in trabecular separation in the AO, AN and A groups. This finding corroborate previous research indicating that alcohol negatively impacts bone by reducing bone biomechanical properties [37]. Moreover, a study conducted by Callaci et al. suggested that ethanol’s effects on bone mass may be attributed to the inhibition of the Wnt signaling pathway, most likely through the stimulation of oxidative stress [46]. Alcohol also exerts its influence at the cellular level by modulating gene expression, including that of bone morphogenetic proteins and osteocalcin, ultimately resulting in the inhibition of bone formation [41].

Despite the significance of alcohol toxicity, studies on its effect on bone remain scarce. Decreased bone mass, a prominent characteristic of osteoporosis, may be attributed to reduced bone formation and/or increased bone resorption, both of which are potentially impacted by ethanol. Santori et al. demonstrated decreased osteocalcin levels in alcoholics, indicating a direct impact on bone metabolism [47]. Alcohol’s effects on bone can manifest directly, affecting the number and activity of osteoblasts and osteoclasts, and contributing to an increase in osteocyte apoptosis [48]. Additionally, alcohol accelerates lipid peroxidation and causes protein modifications, as reported in numerous previous studies, while the administration of antioxidant agents was shown to reduce levels of free iron and inhibit alcohol toxicity [49,50,51]. Moreover, alcohol consumption has been associated with a reduction in volumetric bone mineral density in both men and women [52]. Previous studies have reported that ethanol impairs bone mesenchymal cell differentiation via the suppression of the Wnt/β-catenin pathway [53,54].

Here, we have shown that vitamin E, whether in the form of alpha-tocopherol or palm vitamin E, has the potential to reverse the effects of alcohol and restore bone loss in osteopenic rats. Our findings revealed an increase in osteoblast bone formation and a decrease in osteoclastic bone resorption, as evidenced by increased Ob.S/BS (%), OS/BS (%), OV/BV (%) and decreased Oc.S/BS (%), ES/BS (%). Interestingly, palm vitamin E exhibits superiority over alpha-tocopherol in all parameters except OV/BV (%). The same effects were also observed in structural parameters, where both isoforms of vitamin E treated group increased trabecular thickness, number, and volume after alcohol exposure. This finding is consistent with our laboratory’s previous work by Hermizi et al., which demonstrated that vitamin E at a dosage of 60 mg/kg for 2 months improved the structure and cellular properties of bone in nicotine-induced rats [55]. Furthermore, both palm vitamin E and alpha tocopherol have demonstrated the ability to reverse bone mineral loss induced by alcohol in the same rat model. However, only palm vitamin E was able to improve bone strength in alcohol-treated rats [37]. Vitamin E safeguards bone by inhibiting free radical chain reactions and thwarting lipid peroxidation-induced damage [25]. In addition to its antioxidant properties, palm vitamin E has been shown to regulate bone-related gene expression in nicotine-induced rats [36]. Vitamin E has also been shown to increase bone density and trabecular bone, prevent bone calcium loss, and reduce bone calcium loss in rat [56]. It provides a shield for bones from oxidative damage [57]. Supplementation of vitamin E, together with calcium, enhanced bone formation and suppressed bone loss in ovariectomized rats [58]. Vitamin E enhances bone formation and reduces osteoclasto-genesis via its antioxidant and anti-inflammatory properties through two mechanisms: inhibition of nuclear factor kappa B ligand (RANKL) expression and inhibition of inflammatory cytokine expression [59]. Reddy et al. reported that treatment with vitamin E reversed ethanol-induced lipid peroxidation in alcohol-induced male rats [18].

Comparing vitamin E-treated groups, palm vitamin E demonstrated superior efficacy in improving cellular and structural bone histomorphometric parameters compared to alpha-tocopherol. This finding aligns with previous studies, which have consistently shown palm vitamin E to be more effective than alpha-tocopherol in mitigating bone damage induced by free radicals [60,61,62]. Additionally, tocotrienols have exhibited superiority over alpha-tocopherol in various domains, including their role as potent antioxidant agents in inflammation treatment [63,64], improving biomechanical properties in post-menopausal osteoporosis [65], and enhancing bone biomechanical properties in normal rats [66,67]. The observed effectiveness of palm vitamin E over alpha-tocopherol in this study may be attributed to structural differences between these two forms of vitamin E, specifically tocotrienols’ unsaturated side chain, which allows for better tissue penetration [60] and confers stronger protection against oxidative stress compared to alpha-tocopherol.

While our current study focused on how alpha tocopherol and palm vitamin E impact bone health, it is important to consider the roles of different types of vitamin E, such as gamma tocopherol and delta tocotrienol, in bone metabolism. Our previous work has shown that palm oil vitamin E (which contains the most abundant gamma tocotrienol) improves bone strength and mineral density in alcohol-treated rats [37]. We also observed that gamma tocotrienol (GTT) can enhance the structural and biomechanical properties of normal mice [66].This suggests that different tocotrienol forms could help prevent alcohol induced bone loss by reducing stress and limiting bone resorption. Current findings support this idea by showing that palm vitamin E is more effective than alpha tocopherol at enhancing activity and reducing bone resorption. This indicates that tocotrienols from palm oil might provide protection against bone loss compared to alpha tocopherol alone. Moving forward, it would be beneficial for studies to explore how various vitamin E forms compare in models of alcohol induced osteoporosis. This could lead to an insight, into how vitamin E may impact bone health and its possible benefits in treating osteoporosis.

Despite such promising results, a number of limitations have to be considered in the present study. Firstly, the rat model utilized in the study possesses its drawbacks, although generally it contributes much to the understanding of biological processes. There are considerable differences in metabolism, bone physiology, and overall biology between rodents and humans; therefore, the outcomes of various treatments can also differ accordingly. Thereby, while vitamin E from palm oil may be potent in improving chronic alcohol-induced osteoporosis in rats, more clinical-based studies are prerequisite in order to establish whether similar results in human populations can be replicated on the background of complications underpinning human bone metabolism and the multifactorial nature of osteoporosis in humans. Secondly, the use of H and E staining, while effective for examining overall bone structure, lacks specificity. Immunofluorescence could offer more detailed insights into the cellular and molecular mechanisms at play, particularly in identifying specific cell populations and proteins related to bone metabolism. This could help in better understanding the protective effects of vitamin E on bone health in future studies.

In future studies, it would also be highly beneficial to incorporate turnover markers for bone metabolism, such as CTX-I (C-terminal telopeptide of type I collagen) and P1NP (procollagen type I N-terminal pro-peptide), to provide a more comprehensive understanding of the impact of alcohol and vitamin E treatment on bone dynamics. These biomarkers are widely used to assess bone resorption and formation, respectively, offering insights into the rate of bone turnover and the balance between bone formation and resorption. Additionally, the inclusion of DEXA data would provide further validation of the results by offering a standard, non-invasive technique for assessing bone mineral density (BMD). Since DEXA is commonly used for diagnosing osteoporosis and evaluating bone health, it would be valuable to see how the effects of alcohol and vitamin E correlate with BMD measurements, complementing the histomorphometric analysis and strengthening the overall conclusions regarding the potential of vitamin E in preventing or treating alcohol-induced bone loss.

## 3. Materials and Methods

### 3.1. Animal Experiments

The male Sprague–Dawley rats, 3 months old, weighing between 250 and 300 g, were acquired from the Laboratory Animal Resource Unit, Faculty of Medicine, Universiti Kebangsaan Malaysia. They were maintained on a standard diet and housed in pairs under a 12-h natural light-dark cycle, with ad libitum access to tap water. Ethical clearance for the study was obtained from the Universiti Kebangsaan Malaysia Animal Ethics Committee with approval number FP/FAR/2013/NORAZLINA/17-JULY/529-AUG.-2013-JULY-2015.

### 3.2. Experimental Design

**Figure 12:**
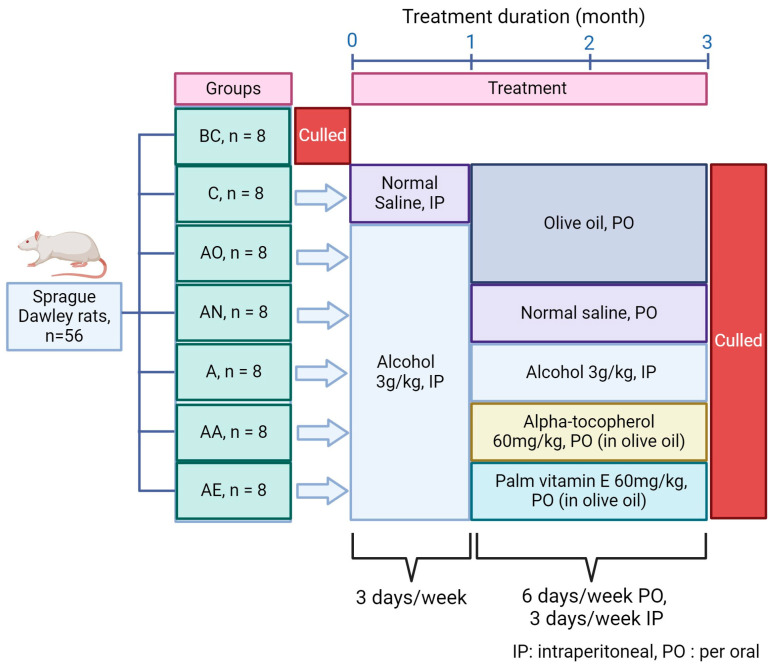
Animal groups and treatment phases. 56 rats were randomly assigned to six different groups (n = 8): Baseline Control (BC), Control (C), Alcohol Olive Oil (AO), Alcohol Normal Saline (AN), Alcohol (A), Alcohol Alpha-Tocopherol (AA), and Alcohol Palm Vitamin E (AE). Alcohol (3 g/kg, 3 days/week, IP) was administered for 1 month to induce bone loss, followed by 2 months of treatment with either olive oil, normal saline, alpha-tocopherol, or palm vitamin E. Alpha-tocopherol and palm vitamin E were given orally (60 mg/kg, 6 days/week). At the end of the treatment, rats were euthanized, and femurs were collected for analysis.

Fifty-six rats were randomly assigned to seven groups, each comprising eight individuals, as illustrated in Figure 12. These groups included baseline control (BC, euthanized without treatment), control (C, receiving normal saline for 1 month, followed by olive oil for 2 months), alcohol olive oil (AO, administered alcohol for 1 month, followed by olive oil for 2 months), alcohol normal saline (AN, exposed to alcohol for 1 month, followed by normal saline for 2 months), alcohol (A, treated with alcohol for 3 months), alcohol alpha-tocopherol (AA, subjected to alcohol for 1 month, followed by alpha-tocopherol for 2 months), and alcohol palm vitamin E (AE, treated with alcohol for 1 month, followed by palm vitamin E for 2 months). Alcohol administration involved intraperitoneal injection at a dosage of 3 g/kg, three times a week. Alpha-tocopherol and palm vitamin E were orally administered via gavage at a dosage of 60 mg/kg, six days a week.

### 3.3. Binge Alcohol Model

Alcohol was given as a single intraperitoneal injection using a 20% ethanol solution mixed with saline, at a dose of 3 g/kg. This dosage was selected to reach peak blood alcohol levels of around 300 mg/dL. Injections were administered on three consecutive days per week, with a volume of 0.4 mL per 100 g of rat body weight. To simulate a binge drinking pattern, no injections were given on the remaining four days of each week. This regimen has been validated to induce bone loss in rats [46,68]. Control animals were subjected to the same stress conditions by receiving intraperitoneal saline injections.

### 3.4. Vitamin E Supplementation

Vitamin E (3 g) (Sime Darby, Malaysia, batch number: SB13112870) or alpha tocopherol (Sigma, Burlington, MA, USA, product number: 258024) was dissolved in 50 mL of olive oil (Bertolli, Lucca, Italy) to create a solution with a concentration of 60 mg/kg of rat body weight. Each rat received 0.1 mL of this solution per 100 g of body weight via oral gavage. This dosage has been shown to restore healthy bone in other osteoporotic rat models [36,59]. Throughout the study, the rats were weighed weekly. At the end of the treatment period, the rats were anesthetized with diethyl ether and subsequently culled. The femora were then extracted, with the distal parts fixed in a 70% ethanol solution for bone histomorphometric analysis.

### 3.5. Bone Histomorphometry

Histomorphometric measurements were taken in the region of the metaphysis, which is abundant in trabecular bone. This area is situated 3–7 mm away from the base of the growth plate and 1 mm from the cortex.

To evaluate cellular parameters, femur bones were decalcified over two months using a 10% ethylenediaminetetraacetic acid (EDTA) solution at pH 7.4. The decalcified bones were then embedded in paraffin wax, sectioned into 6 μm-thick slices using a microtome, and stained with Hematoxylin and Eosin (H and E). The study assessed several cellular parameters, including the osteoid surface relative to bone surface (OS/BS), osteoblast surface relative to bone surface (Ob.S/BS), eroded surface relative to bone surface (ES/BS), osteoclast surface relative to bone surface (Oc.S/BS), and osteoid volume relative to bone volume (OV/BV).

To measure structural parameters, undecalcified bone samples were embedded in polymerized methyl methacrylate. These samples were cut into 9 μm-thick sections using a Leica RM2155 microtome (Leica, Nussloch, Germany) and stained using the Von Kossa method to highlight mineralized bone. The structural histomorphometric parameters analyzed included trabecular thickness (Tb.Th), trabecular number (Tb.N), trabecular separation (Tb.Sp), and trabecular bone volume as a percentage of total volume (BV/TV).

Bone changes were analyzed using an Image Analyzer ECLIPSE 80i (Nikon, Tokyo, Japan) equipped with Pro-Plus 5.0 software (Media Cybernetics, Silver Spring, MD, USA), a Q Imaging camera (Micropublisher 5.0, Toronto, ON, Canada), and a light microscope (Nikon, Tokyo, Japan). The results were expressed utilizing bone histomorphometric measurements as recommended by the American Society of Bone and Mineral Research Histomorphometry Nomenclature Committee [69].

### 3.6. Statistical Analysis

The results are reported as the mean ± standard error of the mean (SEM). Data analysis was conducted using SPSS software (version 20). For data with a normal distribution, one-way ANOVA was used, followed by Tukey’s post hoc test for pairwise comparisons. For data that did not follow a normal distribution, the Kruskal–Wallis test and Mann–Whitney test were applied. Differences were considered statistically significant at a level of *p* < 0.05 for all analyses. 

## 4. Conclusions

**Figure 13:**
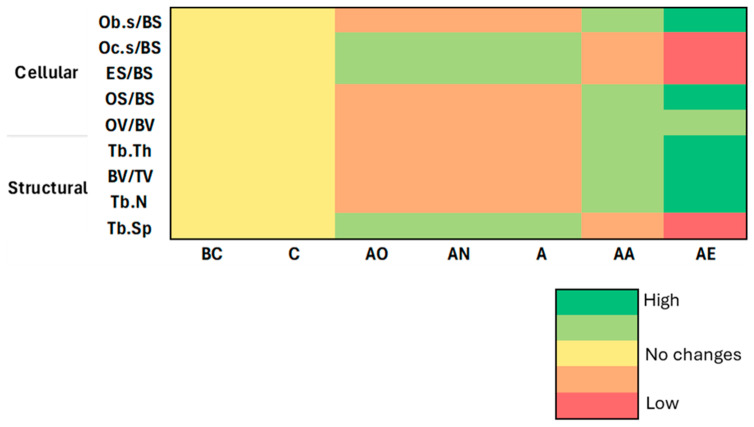
Summary of the results of the vitamin E effect on cellular and structural bone histomorphometry against both controls. Abbreviation: Cellular parameters measured: Ob.S/BS: Osteoblast surface/bone surface, Oc.S/BS: osteoclast surface/bone surface, ES/BS: eroded surface/bone surface, OS/BS: osteoid surface/bone surface, and OV/BV: osteoid volume/bone volume. Structural parameters measured: Tb.Th: trabecular thickness, BV/TV: bone volume to total volume, Tb.N: trabecular number, Tb.Sp: trabecular separation. Treatment groups: BC: Baseline Control, C: Control, AO: Alcohol Olive Oil, AN: Alcohol Normal Saline, A: Alcohol, AA: Alcohol Alpha-Tocopherol, AE: Alcohol Palm Vitamin E.

In summary, our findings (Figure 13) indicate that alcohol induces substantial bone loss at structural and cellular levels, whereas treatment with vitamin E not only reverses these effects but also promotes bone formation beyond control levels. Particularly, palm vitamin E exhibited superior efficacy compared to alpha-tocopherol potentially due to the broader spectrum of tocotrienols present in palm vitamin E. Thus, palm vitamin E appears to be a promising candidate for therapeutic intervention in repairing alcohol-induced bone damage. To better understand the mechanisms underlying these effects, future research should explore on biochemical markers such as Runx2, TRAP, and gene expression markers like Osterix (*SP7*) and Bone Morphogenetic Proteins (BMPs), which will provide deeper insights into the molecular pathways involved.

## Data Availability

All relevant data are presented in this manuscript.
